# Aero-Thermo-Dynamic Mass Analysis

**DOI:** 10.1038/srep28849

**Published:** 2016-07-14

**Authors:** Kota Shiba, Genki Yoshikawa

**Affiliations:** 1 International Center for Young Scientists (ICYS), National Institute for Materials Science (NIMS), 1-1 Namiki, Tsukuba, 305-0044 Ibaraki, Japan; 2 World Premier International Research Center Initiative (WPI), International Center for Materials Nanoarchitectonics (MANA), National Institute for Materials Science (NIMS), 1-1 Namiki, Tsukuba, 305-0044 Ibaraki, Japan; 3 Materials Science and Engineering, Graduate School of Pure and Applied Science, University of Tsukuba, Tennodai 1-1-1 Tsukuba, 305-8571 Ibaraki, Japan

## Abstract

Each gas molecule has its own molecular weight, while such a microscopic characteristic is generally inaccessible, and thus, it is measured indirectly through *e.g.* ionization in conventional mass analysis. Here, we present a novel approach to the direct measurement of molecular weight through a nanoarchitectonic combination of aerodynamics, thermodynamics, and mechanics, transducing microscopic events into macroscopic phenomena. It is confirmed that this approach can provide molecular weight of virtually any gas or vaporizable liquid sample in real-time without ionization. Demonstrations through analytical calculations, numerical simulations, and experiments verify the validity and versatility of the novel mass analysis realized by a simple setup with a flexible object (*e.g.* with a bare cantilever and even with a business card) placed in a laminar jet. Owing to its unique and simple working principle, this aero-thermo-dynamic mass analysis (AMA) can be integrated into various analytical devices, production lines, and consumer mobile platforms, opening new chapters in aerodynamics, thermodynamics, mechanics, and mass analysis.

The discovery of anode rays by Goldstein in 1886 initiated the history of mass analysis, and then, the first mass spectrometer by Thomson in 1912 established the trend of mass analysis based on ionization. It continues even for the advanced matrix assisted laser desorption/ionization method^1^, whereas the ionization process inevitably requires large vacuum-based instrumentation, hindering the technology from contributing to the wider society. For the mass analysis without ionization, advanced nanomechanical technology will be one of the candidates as they demonstrated ultra-high resolution of mass sensing (up to yocto gram detection) without ionization^2^,^3^. However, they again require larger vacuum environment for higher frequency measurements and such resonance-based mass sensing is basically difficult to be applied to common gases with low molecular weights. In addition, common chemical affinity-based sensors, including typical nanomechanical sensors, have to be coated with specific receptor materials to acquire physical and/or chemical information of molecules such as molecular type or mass^4^. Therefore, a new simple approach to the mass analysis without the needs of ionization, bulky instrumentation, vacuum condition, and receptor coating has been highly demanded for decades.

Here we report for the first time that molecular weight of the gas species can be directly measured through the mechanical deformation of a simple elastic object placed under a constant gas flow. The present aero-thermo-dynamic approach to real-time mass analysis in ambient condition requires neither ionization process, bulky instrumentation, nor receptor coating. The capability for miniaturization based on its simple working mechanism provides an opportunity of being integrated into mobile devices such as a smart phone, leading to the contribution to the wider range of fields where conventional mass analysis cannot reach. In the present study, we will demonstrate some applications utilizing the novel approach to the molecular mass analysis of gas/liquid samples along with full verification of its working principle by means of analytical model, finite element analysis, and experiments.

## Results and Discussion

Towards the analytical description of the new aero-thermo-dynamic mass analysis without ionization, here we start with revisiting the classical fields, focusing on their nanoarchitectonic aspects combining microscopic events and macroscopic phenomena. The effective combination of nano and macro can be found in the well-known ideal gas law:


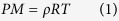


where *P*, *ρ*, *R*, and *T* are related with macroscopic aspects (atmospheric pressure, density of gas, gas constant, and temperature, respectively), while *M* represents microscopic characteristic, that is, molecular weight. Then, we introduce another basic equation known as the “drag equation”^5^,^6^:


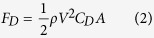


where *F*_*D*_ is drag force, *V* is velocity of gas, *C*_*D*_ is drag coefficient, and *A* is area of the orthographic projection of the object on a plane perpendicular to the direction of motion. All the parameters and typical values used in the present study are summarized in Table S1. The combination of these equations via the common parameter *ρ* gives,


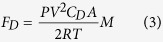


This equation indicates that molecular weight of target gases has a certain relationship with macroscopic force (*F*_*D*_), while the complete analytical expression usually requires the evaluation of *V* and *C*_*D*_ which are also affected by *M* in general (see Supplementary materials for details).

One of the simplest approaches to measuring *F*_*D*_ is the use of a cantilever placed in a gas flow. As described in the Supplementary materials, a classical model in mechanics (Euler-Bernoulli equation)^7^ combined with a three dimensional axially symmetric laminar jet model provides the relationship between *F*_*D*_ and the cantilever deflection which can be easily measured with various methods including optics (*e.g.* laser reflection) and electronics (*e.g.* piezoresistors). The detailed analytical investigation led us to a simple expression of the cantilever deflection at its free end (*z*(0)) as follows:


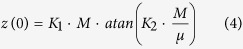


where *K*_1_ and *K*_2_ are constant values determined by the experimental parameters (see details in the Supplementary materials) and *μ* is viscosity coefficient. This analytical model based on aerodynamics, thermodynamics, and mechanics bridges the divide between a microscopic characteristic (molecular weight, *M*) and a directly measurable macroscopic phenomenon cantilever deflection, *z*(0). It is found that the cantilever deflection is determined by a linearity part (*K*_1_·*M*) with an arctangential modulation (*atan*(*K*_2_·*M*/*μ*)). Since the arctangent function gradually approaches to a constant value (π/2) with a larger value of *K*_2_·*M/μ* (*e.g.* >98% of *π*/2 with *K*_2_·*M/μ*  > 35), the deflection of a cantilever can be linearly proportional to molecular weight by tuning the experimental conditions. It should be noted that the simple expression of equation (4) is valid as long as a gas sample with a low Reynolds number forms a laminar jet. For such gas samples, *C*_*D*_ is found to be inversely proportional to square root of the Reynolds number (*Re*), leading to the simple expression as discussed in the Supplementary materials. While the condition for the inverse proportionality depends on geometrical features of sensing elements^5^, most applications will be conducted within a low *Re* region, *e.g. Re* smaller than a few thousands. Further validations of the utilization of the ideal gas law (equation (1)) and the influence of Joule-Thomson effects are also discussed in the Supplementary materials.

**Figure 1:**
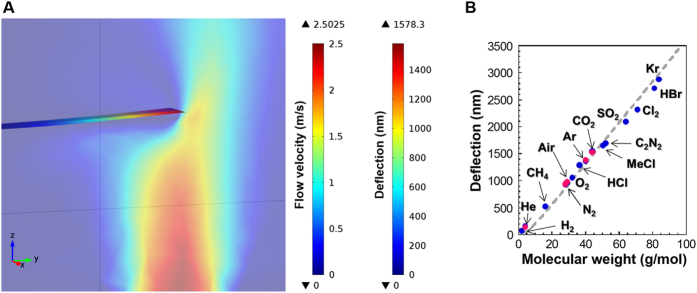
The present mass analysis applicable to various gases. (**A**) Schematic of a gas flow profile in the confined space depicted by FEA simulation. (**B**) Obtained deflection of a cantilever as a function of molecular weight of various molecules (5 for experiments and 15 for FEA simulations). A gray dashed line is drawn based on the analytical calculation. The deflection value was measured at the tip of the cantilever. The results obtained by the experiments and FEA simulations are presented in pink and blue dots, respectively.

To verify the validity of this analytical discussion, we utilized finite element analysis (FEA) along with simultaneous experimental confirmation by means of advanced digital holographic imaging^8^ and 3D-printing technologies. We built a simple model in which a gas flowed toward a cantilever (Fig. 1A). The relationship between molecular weight of 15 different gases and the corresponding deflections was examined, varying all the accessible parameters such as geometric sizes and gas flows. Details on the FEA simulation are presented in the Methods section. We found that the deflection monotonically increases with molecular weight under the present condition as indicated by the analytical model (equation (4)) (Fig. 1B).

**Figure 2:**
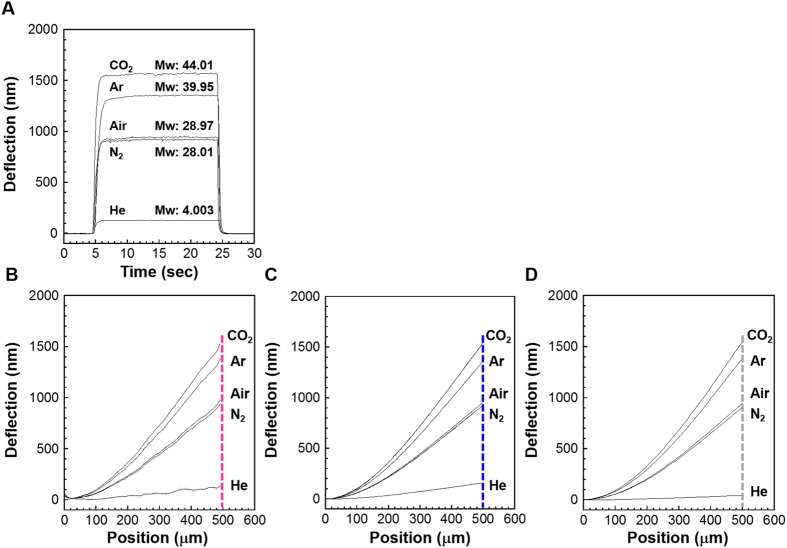
Mechanical transduction of various molecular weights verified by experiments, numerical simulations, and analytical calculations. (**A**) Measured deflection of a cantilever induced by the flow of five gases (helium, nitrogen, air, argon and carbon dioxide) at a flow rate of 6.0 mL/min. (**B**) Deflection profile of a cantilever obtained by experiments using DHM, (**C**) FEA simulations, and (**D**) analytical calculations. Flow rate was set at 6.0 mL/min in all experiments and calculations.

This relationship was also confirmed by the experiments using a digital holographic microscope (DHM; R2100, Lyncee Tec SA) which enables real-time monitoring of vertically moving objects with sub-nanometer precision and the 3D-printed chambers with the same dimensions as those simulated in FEA. Detailed information on the experimental setup is described in Fig. S1. By using a mass flow controller, five common gases including helium, nitrogen, air, argon, and carbon dioxide were flowed into the sealed cantilever-mounted chamber through a hole beneath the cantilever (Fig. S2). The gas exhausted was collected to confirm an exact flow rate with a flow meter placed at the end of the present setup. The motion of the entire cantilever body was measured by DHM through a glass window on top of the chamber (Figs S3 and S4, and Movie S1). As shown in Figs 1B and 2A,B, the relationship between the cantilever deflection and molecular weight was confirmed to be consistent with those derived from the FEA simulations (Fig. 2C) and the analytical model (Fig. 2D). Based on the present setup and conditions, the amount of sample gas, required for molecular weight determination is approx. 0.1 mL (response time: 1 sec. and flow rate: 6 mL/min, Fig. 2A). The sample amount can be further reduced through a comprehensive optimization of the present system.

**Figure 3:**
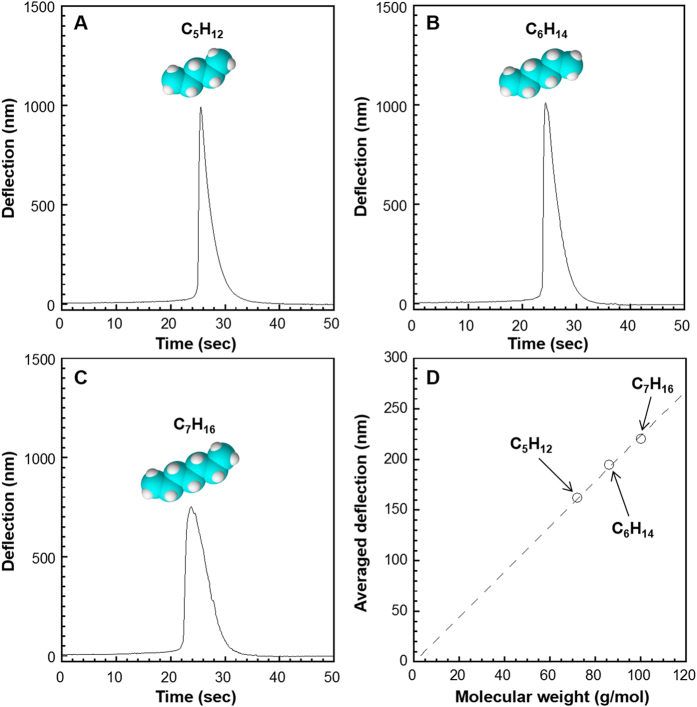
Molecular weight analysis of liquid samples. Deflection peaks induced by vaporized samples of (**A**) pentane, (**B**) hexane, and (**C**) heptane. Each liquid sample was injected into an oven heated at 150 °C. Vaporized samples were then transferred to the cantilever chamber by helium carrier gas and flowed towards a cantilever. (**D**) Averaged deflections derived from each deflection peak as a function of molecular weight.

To demonstrate further versatility of this approach, molecular weights of pentane, hexane, and heptane, which are all in liquid form at room temperature, were examined. Each sample was injected into an oven of a gas chromatography system and heated at 150 °C to vaporize. Then, the vaporized sample was carried by a constant flow of helium gas to the measurement chamber (see Methods for details). The injected liquid samples resulted in peaks of deflection (Fig. 3). The average deflection values calculated for each peak are confirmed to be almost proportional to the molecular weights, following equation (4). These results indicate that molecular weight of vaporizable liquid samples can be also obtained by using the present method.

**Figure 4:**
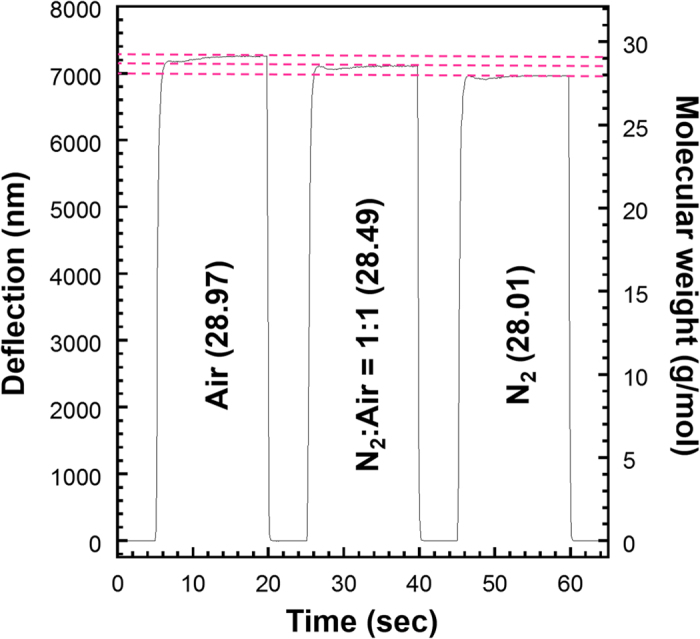
An example of discriminating gases with similar molecular weights. Deflection of a cantilever induced by the flow of air, nitrogen, and mixture of them in equal proportions at the flow rate of 34 mL/min.

To estimate the resolution of the current system, we measured two gases having similar molecular weights, synthesized air (28.97 g/mol) and pure nitrogen (28.01 g/mol), with a larger flow rate (see Methods for details). Based on the tip fluctuation of around 10 nm in the presented measurements, the resolution was estimated to be approx. 0.04 g/mol, which is sufficient to clearly distinguish between nitrogen and air even though the difference in their molecular weights is 0.96 g/mol (Fig. 4). As we indicated in the previous paragraph, the resolution can be further improved by optimizing the experimental parameters according to the analytical model (equations (S10) and (S11) in Supplementary Discussion). A few orders of magnitude improvement would be possible through a comprehensive optimization of the effective parameters, especially those related with sample flow and geometric parameters of a cantilever. For reading out the mechanical deflection in practical applications, the DHM can be replaced by a simple setup, such as capacitive or self-sensing piezoresistive read-out^9^,^10^. With this method, the molecular weight can be determined in a few seconds following transient instability during gas introduction because of turbulence. It should be emphasized here that, in contrast to the conventional mass spectroscopy, this method does not need ionization procedure or vacuum condition which inevitably results in a larger setup.

**Figure 5:**
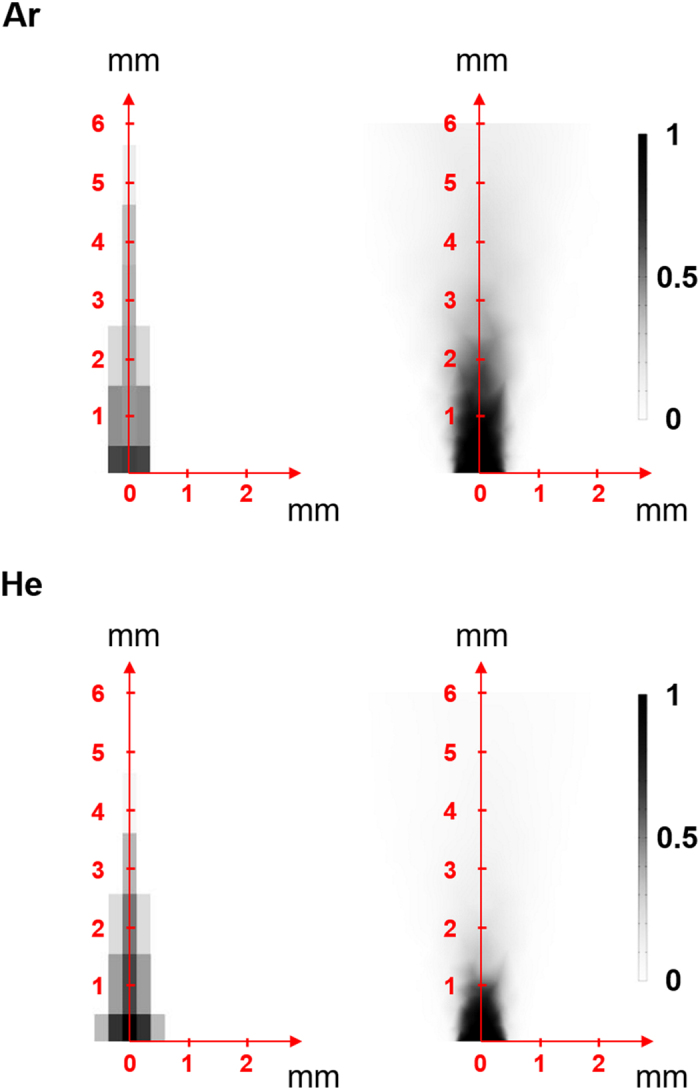
Visualization of helium and argon gas flow profiles in air. Experimental results are shown on the left, and profiles obtained by FEA simulation are shown on the right. Each gas was flowed upwards from (0,0) position with a flow rate of ca. 150 mL/min.

As observed in the case of an air sample, which is a mixture of nitrogen and oxygen, this method gives an averaged value of molecular weights of each component included in a sample gas (Fig. 4). Thus, if all the components and their molecular weights are known, the averaged molecular weight measured with this method provides information on the concentration of each component. To demonstrate an application of this approach, we conducted a visualization of gas flow profiles in ambient condition. Helium or argon gas flowed out from the tip of an injection needle was partially sucked by a piezoelectric pump and introduced into a measurement chamber at 16 mL/min. The sucking position was moved every 0.25 mm and 1 mm in lateral and vertical directions, respectively, to obtain a concentration map at a specific plane. A detailed procedure is explained in Methods and Fig. S5. As can be seen from Fig. 5, gas concentration profiles were clearly visualized by means of the present setup, and the diffusive feature of helium gas was experimentally confirmed; diffusion coefficient of helium in air (0.580 cm^2^/s) is about four times larger than that of argon (0.148 cm^2^/s) under atmospheric pressure at 20 °C^11^. FEA simulations agree well with the experimental results (Fig. 5). Although there are several reports on the visualization of volatile compounds profile such as formaldehyde or ethanol^12^,^13^, the concentration maps were obtained by the fluorescence emitted from specific enzymes, indicating that the reported technique can be applied to a limited number of samples. In contrast, the present approach is applicable to virtually any kind of gas sample, offering lots of opportunities in various applications such as visualizations of exhausted gases from vehicles, rockets or factory chimney, or real-time monitoring of chemical reactions which produce specific gases. To analyze each component in a multicomponent gas, the combination of the present approach and gas chromatography will be effective. The recently developed microfluidics-based miniature gas chromatography systems will be a good option toward a next-generation compact GC-MS system^14^.

**Figure 6:**
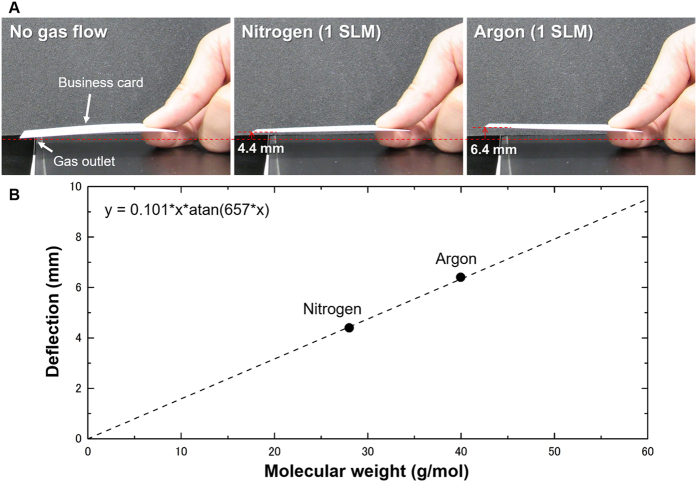
Aero-Thermo-Dynamic Mass analysis in a macroscopic environment. (**A**) Photographs of a business card placed under a constant gas flow. Induced deflections of the business card are visually confirmed. (**B**) Obtained deflections of the business card as a function of molecular weight of nitrogen and argon. The points agree well with an analytically derived dashed line.

We conclude by demonstrating a practical aspect of the present scheme which can be performed with a simple setup. We used a *business card* made of paper and its deflection under a constant gas flow was observed with our eyes. As shown in Fig. 6, the apparent difference in deflection induced by nitrogen and argon was confirmed. All the parameters and typical values used here are summarized in Table S2. The deflection values were found to be proportional to molecular weight and agree well with those derived from the analytical model. This demonstration indicates that any elastic strip fluttering in the wind has been measuring the molecular weight of the air. To achieve a practical device based on the present method, the drag force for the mass analysis can be produced by moving a sensing element, such as a cantilever, instead of flowing a gas sample. One of the examples to realize this kind of situation is to prepare a rotating self-sensing (*e.g.* piezoresistive) cantilever. In such a case, the relative speed of gas, which causes the drag force towards a cantilever, can be controlled by a rotation speed. By using such self-sensing techniques or other simple transduction methods, the present aero-thermo-dynamic mass analysis (AMA) scheme can be integrated into various systems including analytical devices, production lines, and consumer mobile platforms, opening a new era of mass analysis.

## Methods

### Experimental apparatuses and reagents

A digital holographic microscope (DHM) was purchased from Lyncee Tec SA. A mass flow controller (FCST1005C-4F2-F100-N2) was purchased from Fujikin Inc. A gas chromatography-mass spectrometry (GCMS-QP2010 SE) was purchased from Shimadzu Corporation. A flow meter (ProFLOW6000) was purchased from Restek Corporation. A cantilever array chip was purchased from Micromotive GmbH. A cantilever chamber was designed on Autodesk Inventor (Autodesk, Inc.) and prepared using a 3D printer (Objet30 Pro, a product of Stratasys, Ltd.). A piezoelectric pump (Microblower) was purchased from Murata Manufacturing Co., Ltd.

Pentane and heptane were purchased from Nacalai Tesque, Inc. Hexane was purchased from Wako Pure Chemical Industries, Ltd.

### Deflection measurements

For deflection measurements, a mass flow controller was used to control flow rate of gaseous samples including helium, nitrogen, air, argon, and carbon dioxide. Flow rate was controlled in the range from approx. 3.0 mL/min to several tens of mL/min. The mass flow controller was connected to a chamber where a cantilever array chip was mounted. The gas was introduced into the chamber through a hole (0.30 mm in diameter) just beneath a cantilever tip. Distance between the hole and the cantilever tip was 0.45 mm. The chamber was sealed by using two O-rings with different diameters (1.5 mm and 8.0 mm) and a 1 cm square quartz plate with thickness of 2 mm. Details on chamber design are shown in Fig. S2. The introduced gas was exhausted from the outlets and was transferred to a flow meter via a pressure gauge. All the parts were connected with a PTFE tube with 1.0 mm inner diameter (Figs 1B, 2A,B, and 4).

Deflection of a cantilever under gas flow was measured by using the DHM through the quartz plate. The DHM was operated in single laser mode with a wavelength of 683 nm. Region of Interest (ROI) was applied to a single cantilever in order to obtain a stable signal.

### Molecular weight analyses of liquid samples

1 μL of each liquid sample (pentane, hexane, and heptane) was injected into an oven heated at 150 °C with a microsyringe to immediately vaporize the liquid. Then, the vaporized sample was carried by helium gas at ~2.8 mL/min through a capillary column which was heated by a ribbon heater (temperature raised up to ~170 °C). The capillary column was connected to a cantilever chamber heated at ~80 °C. The vaporized sample was flowed through the column toward a cantilever (Fig. 3).

### Sensitivity estimation

Sensitivity was estimated based on the same experimental procedure described above, while the chamber with a hole diameter of 0.50 mm was used. The distance between the hole and a cantilever was set to 0.60 mm. Nitrogen, air and nitrogen/air mixture (1:1 = v/v) were flowed at 34 mL/min (Fig. 4).

### Visualization of gas flow profiles

Helium or argon gas was flowed from bottom up through an injection needle with an inner diameter of 0.70 mm at 150 mL/min (controlled by using a mass flow controller). The gas was then drawn by a PTFE tube which was fixed on an X-Y-Z microscope stage. The tube was first positioned just above the opening and was moved and stopped every 0.25 mm in the lateral direction and every 1 mm in the vertical direction to examine the gas concentration at each point. The gas was sucked by using a piezoelectric pump (Microblower, Murata Manufacturing Co., Ltd.). Flow rate (namely, pumping rate in this case) was maintained at 16 mL/min by adjusting the operation voltage of the Microblower. The relationship between deflection and molecular weight (helium, argon, and air) at 16 mL/min was confirmed in advance. Since the pumped gases should be composed of the two known gases (air and helium, or air and argon), we could determine the mixing ratio of these two components by measuring the deflection which corresponds to the averaged molecular weight. In these cases, the deflection should decrease (increase) by increasing the mixing ratio of helium (argon) because of the smaller (larger) molecular weight compared to that of air (Fig. S5). Thus, concentration of helium (or argon) can be calculated based on the variation from the deflection induced by pure air. The same procedure was repeated for deflection obtained at each point to obtain concentration maps of helium and argon (Fig. 5).

### Finite element analysis (FEA) simulation

To corroborate the validity of the present results in which cantilever deflection is proportional to molecular weight, FEA simulation was performed using COMSOL Multiphysics 4.3. In addition to helium, nitrogen, air, argon, and carbon dioxide, 10 other gases including methane, oxygen, hydrogen chloride, methyl chloride, cyanogen, sulfur dioxide, chlorine, hydrogen bromide, and krypton were also simulated. Each structure was meshed with approx. 100,000 elements, which give sufficient resolution for the present simulations. We modeled the experimental setup with actual dimensions. A cantilever (500 μm × 90 μm × 1 μm) was fixed on the side wall of the chamber (3.00 mm × 2.00 mm × 2.08 mm). The distance between the cantilever and the bottom surface of the chamber was 0.45 mm. The center of a hole with a radius of 0.15 mm was set just beneath the midpoint of the cantilever tip. To simulate a cantilever made of Si single crystal, an elastic tensor was utilized with nine independent parameters: *c*_11_, *c*_12_, *c*_22_, *c*_13_, *c*_23_, *c*_33_, *c*_44_, *c*_55_, and *c*_66_. The corresponding values for these parameters are 160 GPa, 64 GPa, 160 GPa, 64 GPa, 64 GPa, 160 GPa, 80 GPa, 80 GPa, and 80 GPa, respectively (Figs 1A,B, 2C, and 5).

To simulate flow profiles of helium and argon in air (shown in Fig. 5), FEA simulation was also performed using COMSOL Multiphysics 4.3. Each structure was meshed with approx. 40,000 elements, which were found to provide sufficient resolution. A cube with 1.5 cm on a side with a hole of 0.7 mm in diameter was modeled. Molecular weights of helium, air, and argon, densities of helium and argon, and diffusion coefficients^11^ of helium and argon in air were set as 0.004003 kg/mol, 0.028966 kg/mol, 0.03995 kg/mol, 0.1785 kg/m^3^, 1.784 kg/m^3^, 0.58 × 10^−4^ m^2^/s, and 0.148 × 10^−4^ m^2^/s, respectively.

## Additional Information

**How to cite this article**: Shiba, K. and Yoshikawa, G. Aero-Thermo-Dynamic Mass Analysis. *Sci. Rep.* **6**, 28849; doi: 10.1038/srep28849 (2016).

## Supplementary Material

Supplementary Information

Supplementary Movie S1
