# Supplementary material for: Aero-Thermo-Dynamic Mass Analysis
Source: Sci Rep. 2016 Jul 14;6:28849. doi: 10.1038/srep28849 (PMC4944150; doi:10.1038/srep28849)
Supplement: Supplementary Information [file srep28849-s1.pdf]

Aero-Thermo-Dynamic Mass Analysis

Kota Shiba[1,2]* and Genki Yoshikawa[2,3]*

[1]International Center for Young Scientists (ICYS), National Institute for Materials Science (NIMS), 1-1 Namiki, Tsukuba, Ibaraki 305-0044, Japan

[2]World Premier International Research Center Initiative (WPI), International Center for Materials Nanoarchitectonics (MANA), National Institute for Materials Science (NIMS), 1-1 Namiki, Tsukuba, Ibaraki 305-0044, Japan

[3]Materials Science and Engineering, Graduate School of Pure and Applied Science, University of Tsukuba, Tennodai 1-1-1 Tsukuba, Ibaraki 305-8571, Japan

*Correspondence to: SHIBA.Kota@nims.go.jp, YOSHIKAWA.Genki@nims.go.jp

Supplementary Discussion

Validity to utilize the ideal gas law

The effects of molecular volume and the van der Waals interaction between molecules must be considered because the ideal gas law (equation (1) in the main text) does not account for such effects. According to the analyses of the van der Waals equation (*i.e.* the modified ideal gas law for real gases), deviations from the ideal gas law due to these factors were smaller than 0.4%. Therefore, all the gases used in the present measurements can be regarded as ideal gases within this error range. Quantitative discussion is given in the following.

If we handle any type of real gases, we need to consider two factors which are ignored in the case of ideal gases: molecular volume and van der Waals interaction between molecules. The following equation is the well-known van der Waals equation;

$$(P + \frac{an^2}{V_{gas}^2})(V_{gas} - nb) = nRT \tag{S1}$$

where P is atmospheric pressure, V_{gas} is volume of the gas, n is amount of the gas in moles, R is the gas constant, T is temperature, a and b are the van der Waals constants that vary from gas to gas. The values of the van der Waals constants for the gases used in the present study are given in the table below.

	He	N_2	Air	Ar	CO_2
a	0.0340	1.39	1.33	1.35	3.60
b	0.0238	0.0392	0.0366	0.0322	0.0428

Based on these values and the van der Waals equation, we can derive molecular volume of each gas at the atmospheric pressure (1.0 atm). The results are summarized in the table below.

Pressure (atm)	Molecular volume (L)					
	He	N_2	Air	Ar	CO_2	Ideal gas
1.0	24.501 (0.094%)	24.461 (-0.069%)	24.460 (-0.074%)	24.455 (-0.094%)	24.373 (-0.43%)	24.478

The error from the ideal gas can be estimated as 0.094%, -0.069%, -0.074%, -0.094%, and -0.43% for He, N_2, Air, Ar, and CO_2, respectively. Although these values can directly affect the deflection of a cantilever, the error is smaller than the noise level in most cases; for example, the error is ~5 nm at most for the deflection of 1000 nm, while the observed noise value is ~10 nm. Accordingly, the ideal gas law can be applied in the present study as a reasonable approximation.

Discussion on the compressibility of gases and the Joule-Thomson effect

Another factor, which may affect the present results, is the Joule-Thomson effect, whereby the temperature of a sample gas is affected when a compressible gas flows through a valve or a porous membrane. In the presented data, all the gases can be regarded as incompressible because the estimated Mach number of each gas (0.001 – 0.006) is much lower than 0.3, which is a boundary between compressible and incompressible gases. As a result, the Joule-Thomson effect is almost negligible. These conditions can be estimated as follows.

Compressibility of a gas sample can be evaluated by a Mach number of the gas. Mach number, *Ma*, is defined as follows;

$$Ma = \frac{v_f}{v_s} \tag{S2}$$

where v_f is the velocity of fluid and v_s is the speed of sound. The speed of sound is different in each gas and its value is given by the following equation;

$$c = \sqrt{\frac{\gamma RT}{M}} \tag{S3}$$

where γ is heat capacity ratio of gas, *R* is the gas constant, *T* is temperature, and *M* is molecular weight of gas. The values of γ and *M* for each gas are summarized in the table shown below.

	He	N_2	Air	Ar	CO_2
γ	1.66	1.40	1.40	1.67	1.29
M (g/mol)	4.003	28.01	28.97	39.95	44.01

Taking into account the inlet diameter of 0.30 mm in the experimental chamber, the flow volumes of 4.0 mL/min to 7.0 mL/min correspond to the flow velocities of 0.94 m/s to 1.65 m/s. Based on these values, the speed of sound and Mach number of each gas can be estimated as follows;

	He	N_2	Air	Ar	CO_2
v_s (m/s)	970	337	331	308	258
Ma	0.00146	0.00420	0.00428	0.00460	0.00549

Since it is known that a gas with Mach number below 0.3 can be regarded as an incompressible gas, all the gases used in the present study can be treated as incompressible fluids. Consequently, there should be no significant contribution by the Joule-Thomson effect, which takes effect when a compressible fluid flows through a valve or a porous membrane.

<u>Analytical verification of the linearity between drag force, F_D, and molecular weight, M</u>

To derive an analytical expression, we introduce two additional basic models, laminar jet and Euler-Bernoulli beam theory, which will be discussed in the following.

As reported in a previous paper (1), V of a three dimensional axially symmetric laminar jet is given by the following equation.

$$V = \frac{3}{8\pi}\left(\frac{K}{\nu H}\right)\frac{1}{\left[1+\left(\frac{1}{4}\right)\xi^2\right]^2} \tag{S4}$$

where ξ, K, and ν can be expressed as follows;

$$\xi = \frac{1}{4}\sqrt{\frac{3}{\pi}}\frac{\sqrt{K}}{\nu}\frac{x}{H},\ K = \frac{J}{\rho},\ \nu = \frac{\mu}{\rho}$$

H is distance measured along the jet axis from the origin of the jet, x is distance measured perpendicular to the jet axis from the origin of the jet, and J is the axial momentum flux across any

plane normal to the axis of the jet. This laminar jet velocity V will be inserted into equation (3), while V varies depending on the position x. Accordingly, the drag force F_D induced on the cantilever also varies with x.

$$F_D = \frac{9wC_D LM^3P^3Q^4}{8\pi^4\mu^2 l^4 H^2 R^3 \left(\frac{3x^2M^2P^2Q^2}{16\pi^2\mu^2 l^2 H^2 R^2 T^2} + 1\right)^4 T^3} \tag{S5}$$

where w is width of a cantilever, L is length of a cantilever, Q is flow rate, μ is dynamic viscosity of fluid, and l is characteristic length (comparable to width of a cantilever in the present case), respectively.

Now the only one remaining unknown parameter is C_D. It is known that the C_D value varies depending on the shape of objects placed in the fluid stream, and it is frequently discussed with the Reynolds number, Re, defined as the ratio of inertial forces to viscous forces,

$$Re = \frac{\rho V l}{\mu} \tag{S6}$$

where, ρ is density of fluid and V is velocity of fluid, respectively. The C_D value has been known to exhibit complicated variation depending on Re. Although it is difficult to formulate a general expression for the entire Re values, it is possible to approximately describe a specific relationship within a limited range of Re values which is applicable to the laminar gas flows examined in the present study. By plotting the experimentally obtained C_D values and corresponding Re, we found that the following condition between C_D and Re was established,

$$C_D = \frac{\alpha}{\sqrt{Re}} \tag{S7}$$

where α was experimentally estimated to be approx. 5.6.

Finally, to derive a complete analytical expression of the cantilever deflection, the variable drag force F_D together with equations (S6) and (S7) is inserted into the Euler-Bernoulli equation as follows;

$$EI\frac{d^4z}{dx^4} = q = \frac{F_D}{L} \tag{S8}$$

where E is the Young's modulus of a cantilever, I is the second moment of area of the cantilever's cross-section, z is the deflection of the cantilever, and q is one dimensional load applied on the cantilever, respectively. As the cantilever is placed above the origin of jet aligning its free-end with the center of jet, x varies from zero at the free-end to L at the clamped end. The relationship between z and M was obtained by solving the differential equation (S8);

$$\begin{aligned} z(x) = \Big[&-16\pi^2\alpha\mu^2 l^2 wxH^2\,\{\mathrm{atan}(\beta L) - \mathrm{atan}(\beta x)\}\,R^2T^2 - 4\sqrt{3}\pi\alpha\mu lwx \\ &H(x-L)MPQRT - 3\alpha w\left\{(3x-2L)L^2\,\mathrm{atan}(\beta L) - x^3\,\mathrm{atan}(\beta x)\right\} \\ &M^2P^2Q^2\Big] \,/\, \Big[2^{9/2}\pi^2 l^{5/2}E\sqrt{H}IMPRT\Big] \end{aligned} \tag{S9}$$

where β is defined as follows:

$$\beta = \frac{\sqrt{3}MPQ}{4\pi\mu lHRT} \tag{S10}$$

The results are plotted in Fig. 2*D*. As described in the main text, these analytically derived curves correspond well with the curves obtained by experiments and FEA presented in Figs. 2B and 2C.

Then, cantilever deflection at its free end is given by inserting $x = 0$, resulting in the following equation.

$$z(0) = \frac{3\alpha wL^3\,\mathrm{atan}(\beta L)MPQ^2}{2^{7/2}\pi^2 l^{5/2}E\sqrt{H}IRT} \tag{S11}$$

This equation can be simply written as the following form:

$$z(0) = K_1 \cdot M \cdot atan(K_2 \cdot \frac{M}{\mu}) \qquad \text{(S12 and 4 in the main text)}$$

where K_1 and K_2 are constant values determined by the experimental parameters and μ is viscosity coefficient. By inserting all the known parameters into equation (S12), the resolution of analyzed mass can be estimated.

Taking account of the shape of an arctangential curve (refer to the following image), the relationship indicates that the deflection monotonically increases as a function of molecular weight, thereby giving a unique deflection value for each molecular weight.

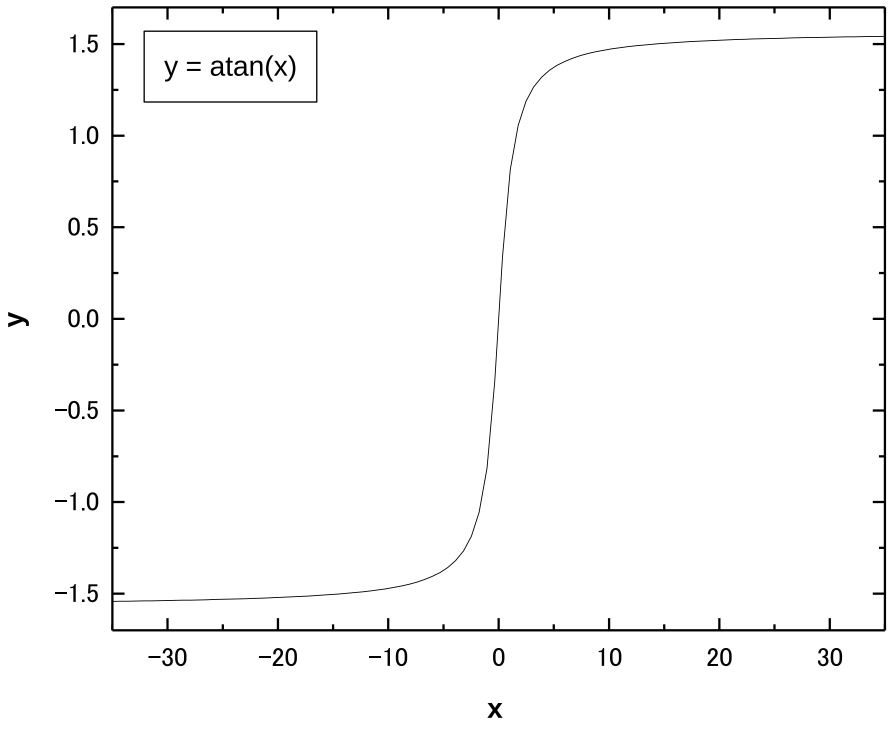

The resolution of analyzed mass is determined by K_1 and K_2 which can be modulated by tuning experimental conditions.

Discussion on the effect of fluid dynamic parameters (drag coefficient C_D and Reynolds number *Re*) on cantilever deflection

As described, C_D values can be estimated from equation (3) using deflection values obtained by the present experiments. The values are summarized in the Table below.

	He	N_2	Air	Ar	CO_2
C_D	29.75	1.565	1.519	1.075	0.9702

According to classical fluid dynamics, C_D correlates with *Re*. Since the relationship between C_D and *Re* is difficult to analytically derive, it is usually determined on the basis of experiments or numerical simulations. It is known that a C_D value decreases as a *Re* value increases up to approx. 10^3 in typical conditions, such as a flow towards a cylinder. To confirm this trend in the present case, we estimated the *Re* values, which can be calculated by equation (S6). In the present case, estimated *Re* values are as follows.

	He	N_2	Air	Ar	CO_2
Re	0.2512	13.78	14.17	22.09	40.49

As shown in the following figure, it is confirmed that the relation between C_D and *Re* follows the typical trend.

Taking advantage of the fact that C_D and Re values are discrete for each sample, we can determine molecular weight M of almost any kind of gaseous and liquid samples even if the linear relationship between cantilever deflection and molecular weight presented in this study is not applicable for some fluid dynamic reasons. The present phenomena occurred in the chamber are affected by several fluid dynamic parameters such as viscosity and/or diffusion. Thus, the linear relationship between

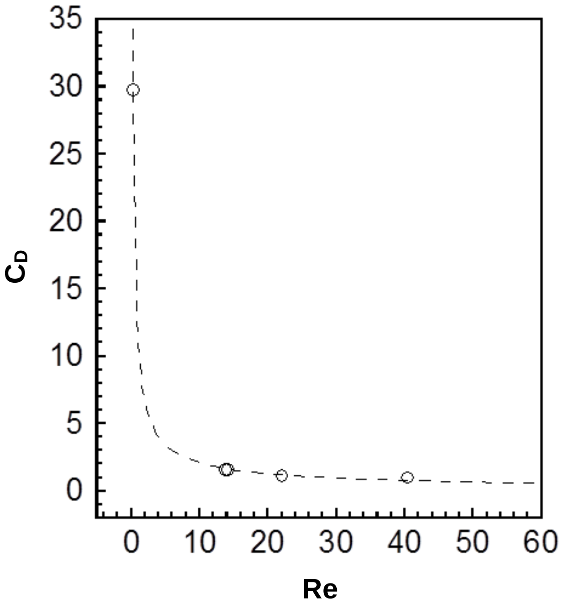

deflection and molecular weight is realized in a limited condition where the contribution from each parameter is well-balanced. However, molecular weight can be still obtained with equation (3) in the main text even in the cases without this linear relationship if the relationship between C_D and V for each sample is known.

Comparison between the present approach and conventional mass spectroscopy

Since the working principle of the presented approach is different from that of the conventional mass spectroscopy, the scopes and the potential applications are also different each other. The characteristics of each approach are summarized in the following table.

		Ionization-based Conventional Mass Spectroscopy (MS)	Aero-Thermo-Dynamic Mass Analysis (AMA)
1	Direct Measurement of molecular mass	**No**	**Yes**
2	Combination with gas chromatography for multi-component gases	*Optional* (integrated spectrum without GC)	*Optional* (averaged molecular mass without GC)
3	Discrimination of structural isomer	**Yes**	**No**
4	Operation in ambient condition	**No** (usually vacuum required)	**Yes**
5	Measurement in real-time	**Yes**	**Yes**
6	Miniaturization	**Difficult**	**Easy**

The explanations on each feature are as follows (Note that the "Ionization-based Conventional Mass Spectroscopy" and "Aero-Thermo-Dynamic Mass Analysis" are abbreviated as "MS" and "AMA"):

1. Direct Measurement of molecular mass

This is the most important feature in AMA, which allows direct measurement of the fundamental property of gas samples, that is, molecular mass. Although MS can identify the gas species by referring to the mass spectrum library, MS cannot measure molecular mass directly. Thus, the integration of AMA into MS system will add another independent parameter (molecular mass), contributing to improving the accuracy of mass spectrum pattern matching.

2. Combination with gas chromatography

In the cases of multi-component gases, gas chromatography is an effective solution for AMA as well as MS. Since AMA can be easily miniaturized, the combination with a compact gas chromatography will realize a mobile mass spectrometer. By integrating AMA into gas chromatography systems, the information on molecular mass will be provided for each peak, achieving comprehensive analysis of multi-component gases without complicated instrumentation related to vacuum system.

In addition to this approach, *stand-alone* operation without gas chromatography is still possible for both MS and AMA. For multi-component gases, MS and AMA provide "integrated spectrum" and "averaged molecular mass", respectively. In both cases, however, it is usually difficult to obtain useful information. The information on sample gases will provide effective solution to compensate the missing parameters. This is demonstrated in the present study by visualizing gas flow profiles. In this case, the gas species are known in advance, and thus, the concentration of each component can be extracted. This approach can be applied to various practical applications, such as monitoring of chemical reaction, environment condition, and exhausted gases from a factory chimney, in which possible chemical species are usually known in advance.

3. Discrimination of structural isomer

This is a major disadvantage of AMA stemming from its working principle, while MS can identify almost any structural isomer according to the mass spectrum library for each chemical specimen. To overcome this problem, we are working on the separation of isomers through the difference in dynamic properties, whereas it is beyond the scope of the present study.

4. Operation in ambient condition

This is another important feature of AMA in contrast to MS which inevitably requires vacuum

condition.

5. Measurement in real-time

For both MS and AMA, the real-time measurement is basically possible. In the case of AMA, this feature becomes more important to measure averaged molecular mass, especially for monitoring purposes. Further, mobile applications will also require the real-time measurements to gain instant response for smell identification, *etc*. As for MS, the real-time measurements may not be an issue for most applications because the separation techniques such as gas chromatography are usually combined.

6. Miniaturization

As demonstrated by a paper strip (actually, a *business card*), AMA does not require any complicated instrumentation for mass analysis. Thus, the first effective and practical application of AMA would be the combination with a compact gas chromatography as mentioned above in "*2. Combination with gas chromatography*". Further, as describe in the conclusion paragraph in the main text, the integration of a mechanics-electronics transduction element (*e.g.* piezoresistors) into a self-moving structure will realize a tiny mass analysis module. Such a module can be technically integrated into a mobile phone. Thus, mass analysis will be no longer for specialized researchers but for anybody, anytime, and anywhere, in the near future based on AMA.

Reference

1. Schlichting H (1933) Laminare Strahlausbreitung. *Ztschr. f. Angew. Math. und Mech.* 13:260-263.

Supplementary Movie

Supplementary Movie S1

Real-time observation of a cantilever deflection using a digital holographic microscope. The cantilever deflection was observed under the flow of various gases.

Supplementary Figures & Tables

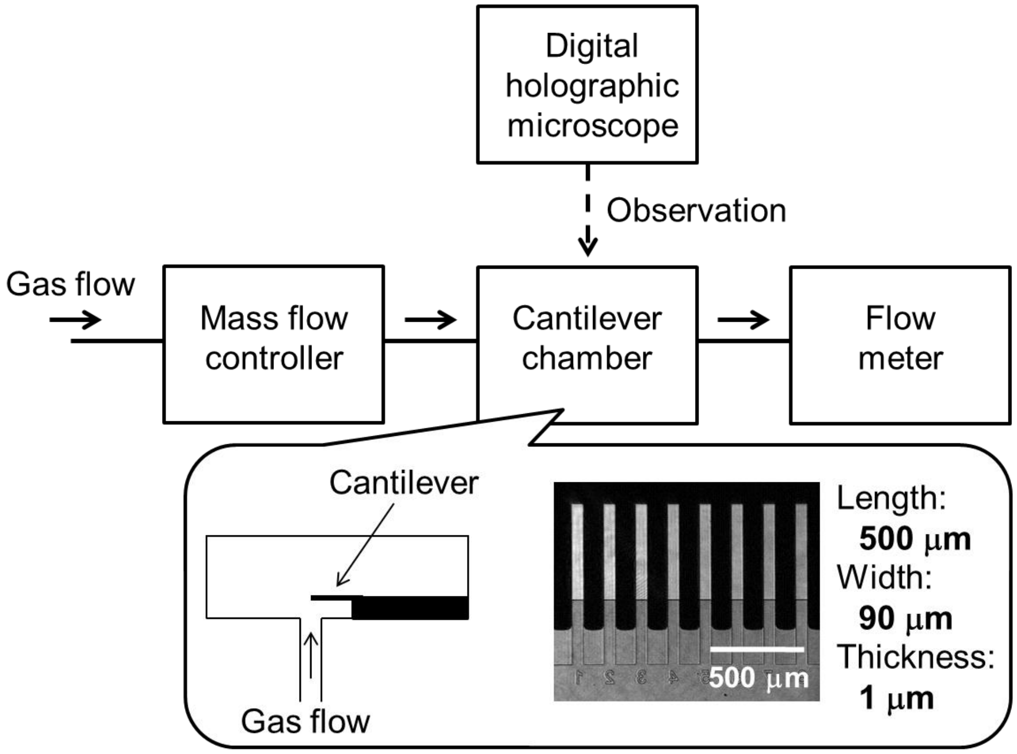

Fig. S1

Schematic of an experimental setup used in the present study.

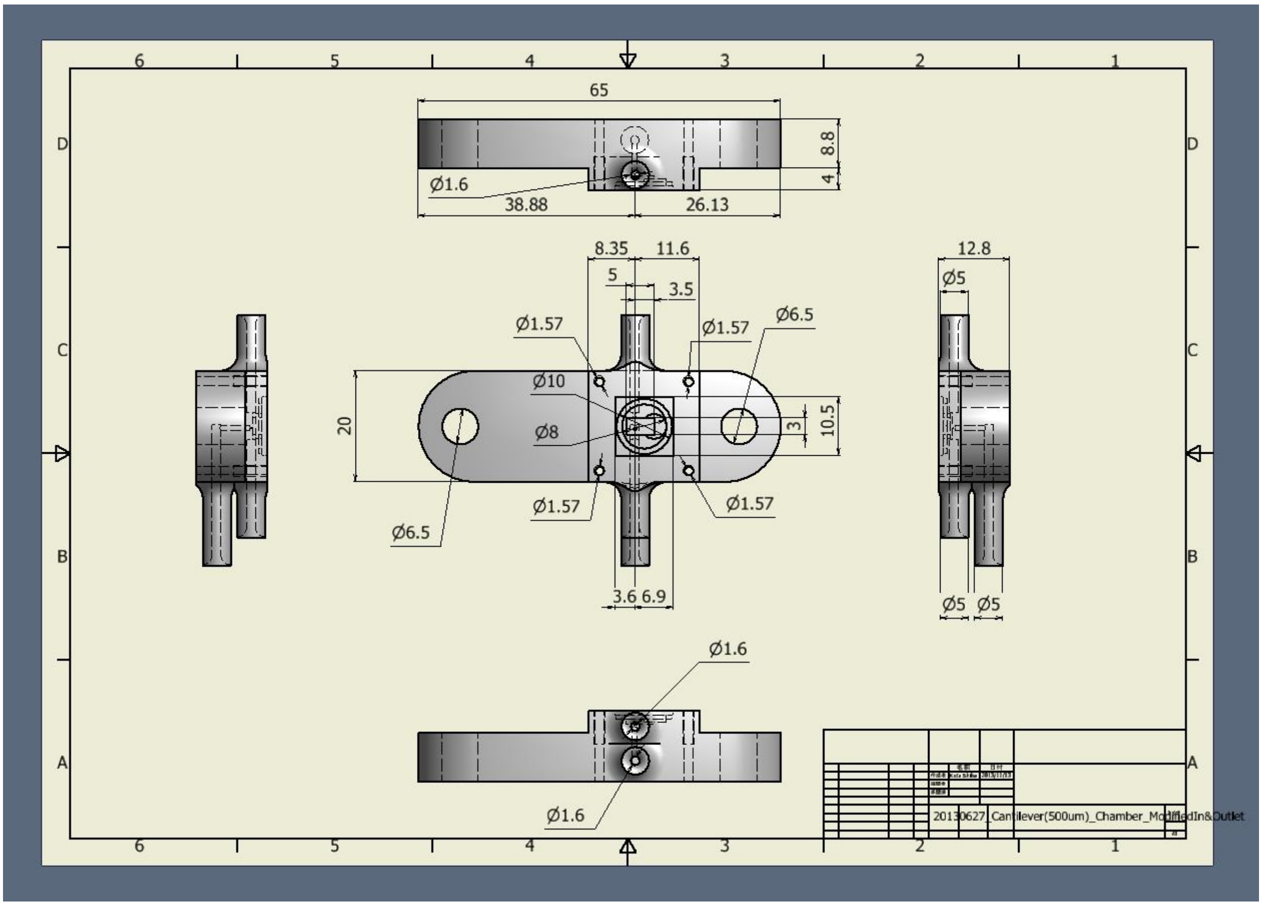

Fig. S2

Details on the cantilever-mounted chamber. One of the CAD software, Autodesk Inventor Professional 2012, was used to draw this figure. All the details on the software can be found in the following link; http://www.autodesk.co.jp/products/inventor/overview.

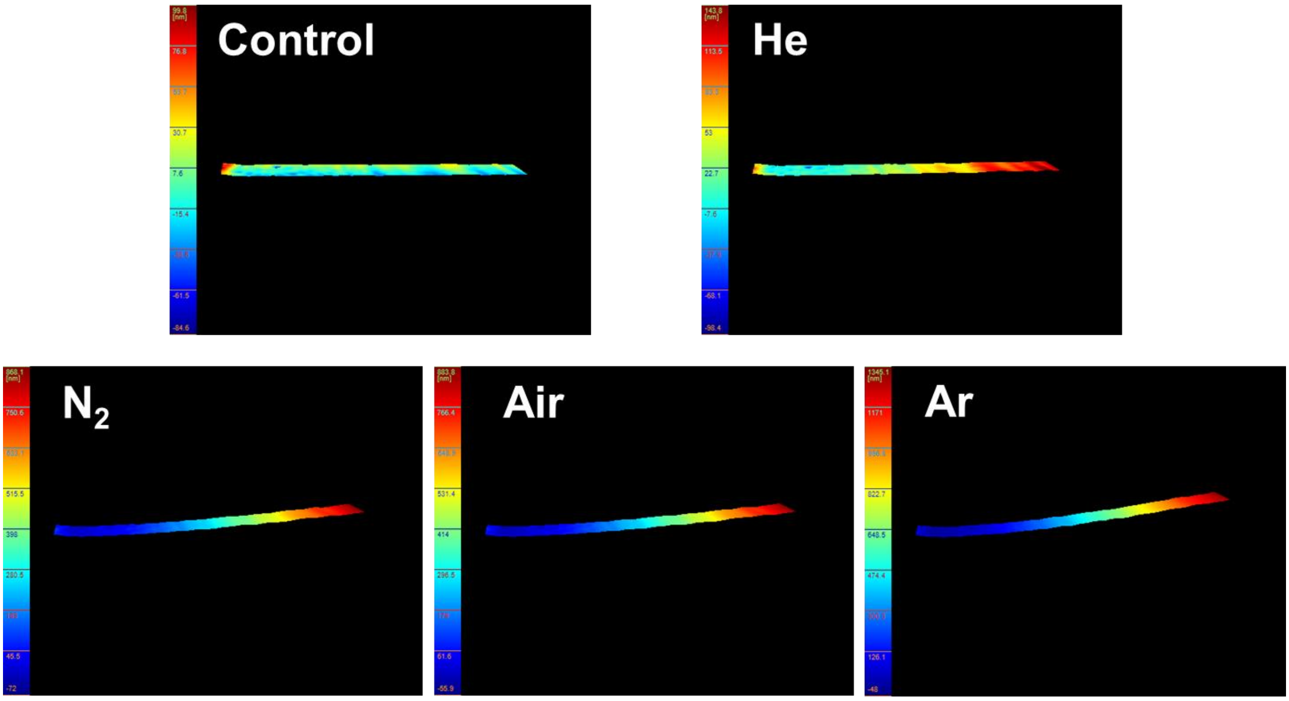

Fig. S3

3D images of a cantilever observed by DHM under a flow of different gases.

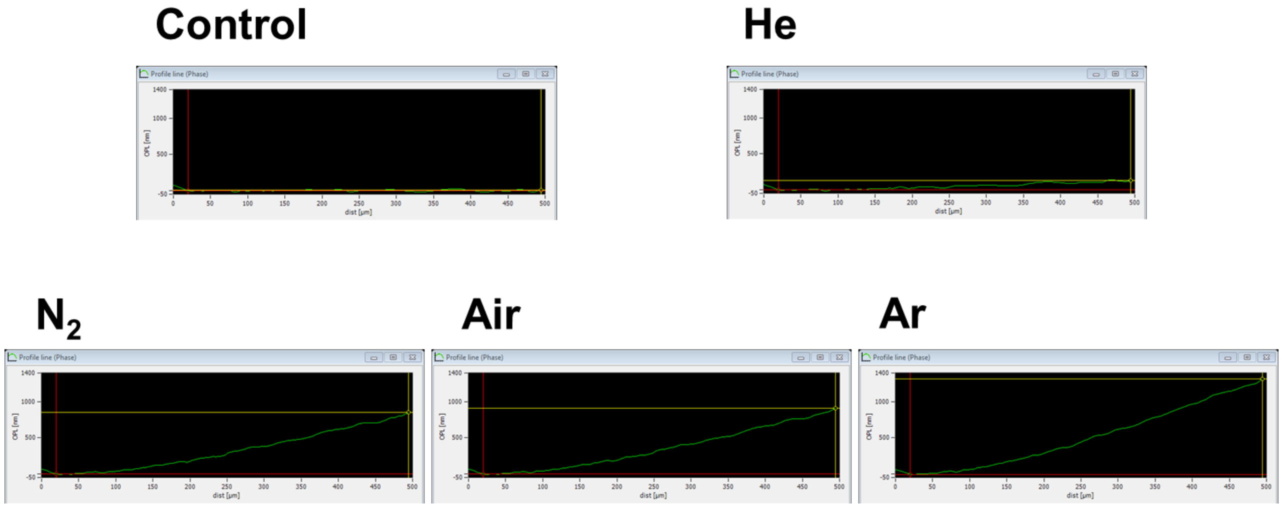

Fig. S4

Line profile (side view) of a cantilever observed by DHM under a flow of different gases.

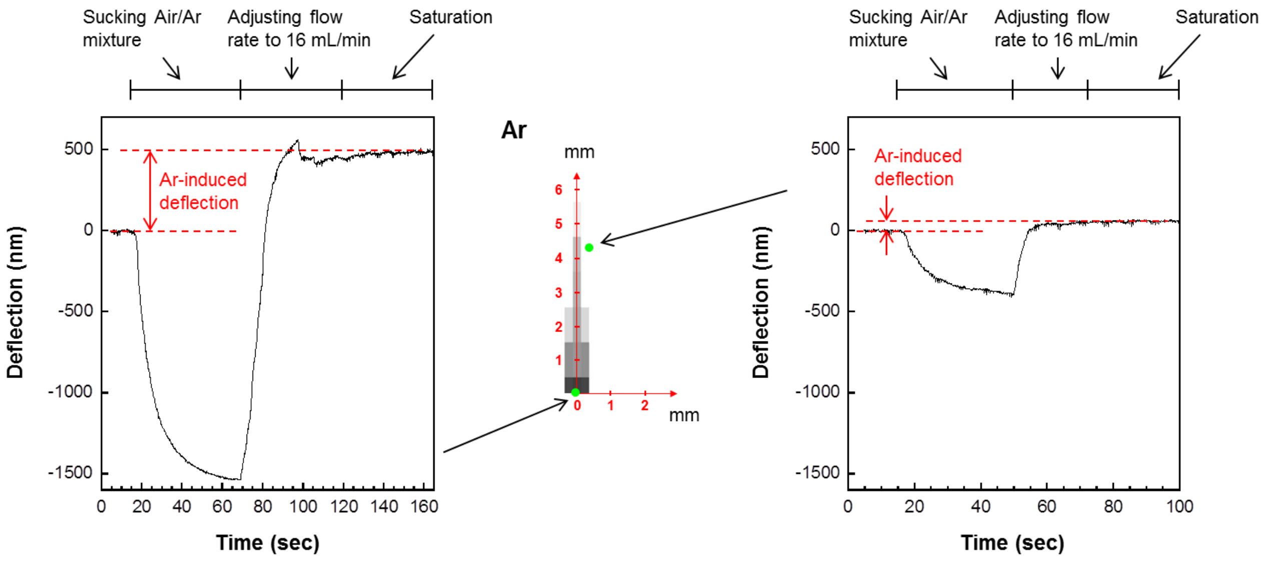

Fig. S5

Examples of cantilever deflection for depicting an Ar concentration map. For the first 20 seconds, we sucked only air with a piezoelectric pump. Deflection caused by air was set as a baseline. When air/Ar mixture was sucked, the amount of gas sucked by the pump changed because of the different density of the gas, leading to the drop of deflection. Then, we adjusted flow rate to the initial value (16 mL/min) by varying the operation voltage of the piezoelectric pump to estimate the increase in deflection which was caused by mixing Ar.

Table S1

Summary of the descriptions and values of the constants used in the present paper.

Description	Symbol	Unit	Value
Drag coefficient	C_D	-	
Drag force	F_D	N	
Area	A	m^2	(w*L)
Density of gas	ρ	kg m^{-3}	
Velocity of gas	V	m s^{-1}	
Reynolds number	Re	-	
Dynamic viscosity	μ	N s m^{-2}	1.84×10^{-5}
Diameter of a pipe	l	m	300×10^{-6}
Atmospheric pressure	P	N m^{-2}	1.01×10^{5}
Molecular weight	M	kg mol^{-1}	
Gas constant	R	J mol^{-1} K^{-1}	8.31
Temperature	T	K	298
Distance measured along the jet axis from the origin of the jet	H	m	450×10^{-6}
Distance measured perpendicular to the jet axis from the origin of the jet	x	m	
Axial momentum flux across any plane normal to the axis of the jet	J	kg m s^{-1}	
Young's modulus of a cantilever	E	N m^{-2}	170×10^{9}
Second moment of area of a cantilever	I	m^4	7.5×10^{-24}
Deflection of a cantilever tip	z	m	
Length of a cantilever	L	m	500×10^{-6}
Width of a cantilever	w	m	90×10^{-6}
Thickness of a cantilever	t	m	1.0×10^{-6}
Flow rate	Q	m^3 s	1.0×10^{-7}

Table S2

Summary of the descriptions and values of the constants used in the experiment with a business card.

Description	Symbol	Unit	Value
Drag coefficient	C_D	-	
Drag force	F_D	N	
Area	A	m^2	(w*L)
Density of gas	ρ	kg m^{-3}	
Velocity of gas	V	m s^{-1}	
Reynolds number	Re	-	
Dynamic viscosity	μ	N s m^{-2}	1.84×10^{-5}
Diameter of a pipe	l	m	700×10^{-6}
Atmospheric pressure	P	N m^{-2}	1.01×10^{5}
Molecular weight	M	kg mol^{-1}	
Gas constant	R	J mol^{-1} K^{-1}	8.31
Temperature	T	K	298
Distance measured along the jet axis from the origin of the jet	H	m	1.00×10^{-3}
Distance measured perpendicular to the jet axis from the origin of the jet	x	m	
Axial momentum flux across any plane normal to the axis of the jet	J	kg m s^{-1}	
Young's modulus of a cantilever	E	N m^{-2}	7.0×10^{9}
Second moment of area of a cantilever	I	m^4	2.67×10^{-14}
Deflection of a cantilever tip	z	m	
Length of a cantilever	L	m	90×10^{-3}
Width of a cantilever	w	m	55×10^{-3}
Thickness of a cantilever	t	m	0.18×10^{-3}
Flow rate	Q	m^3 s	1.67×10^{-5}
